# Chatbots and COVID-19: Taking Stock of the Lessons Learned

**DOI:** 10.2196/54840

**Published:** 2024-03-21

**Authors:** Virginia Arnold, Tina D Purnat, Robert Marten, Andrew Pattison, Hebe Gouda

**Affiliations:** 1 Department of Social Determinants of Health Division of UHC Healthier Populations World Health Organization Geneva Switzerland; 2 Department of Epidemic and Pandemic Preparedness and Prevention, Emergencies Programme World Health Organization Geneva Switzerland; 3 University of Memphis School of Public Health Memphis, TN United States; 4 Alliance for Health Policy and Systems Research Science Division World Health Organization Geneva Switzerland; 5 Department of Digital Health and Innovation Science Division World Health Organization Geneva Switzerland; 6 Department of Health Promotion Division of UHC Healthier Populations World Health Organization Geneva Switzerland

**Keywords:** chatbots, COVID-19, health, public health, pandemic, health care

## Abstract

While digital innovation in health was already rapidly evolving, the COVID-19 pandemic has accelerated the generation of digital technology tools, such as chatbots, to help increase access to crucial health information and services to those who were cut off or had limited contact with health services. This theme issue titled “Chatbots and COVID-19” presents articles from researchers and practitioners across the globe, describing the development, implementation, and evaluation of chatbots designed to address a wide range of health concerns and services. In this editorial, we present some of the key challenges and lessons learned arising from the content of this theme issue. Most notably, we note that a stronger evidence base is needed to ensure that chatbots and other digital tools are developed to best serve the needs of population health.

## Introduction

Like many areas of technology, digital innovation in health is evolving rapidly [1]. It is frequently used to expand access to health services for populations whose options are otherwise restricted due to geographical, economic, or sociocultural barriers. Digital technology has been increasingly used in health care and public health to help disseminate important health-related information to the public as well as health care workers, and to support health services by scheduling medical appointments, for example, or even helping practitioners with the diagnosis of health problems and monitoring health outcomes. During 2020 to 2023, the COVID-19 pandemic led to increased use of digital technology as countries limited in-person access to health care facilities, paused or slowed immunization and other health campaigns, and reallocated health resources to the COVID-19 response. Suddenly, access to health care and essential health services was drastically reduced at a time when health care needs and concerns were increasing.

As a result, there was a tremendous surge of interest in digital technology, including chatbots—computer programs that present a conversation-like interface through which people can access information and services [2]. During the early stages of the COVID-19 pandemic, national and subnational governments, as well as international health organizations such as the World Health Organization (WHO) [3,4] and the United States Centers for Disease Control and Prevention (CDC) [5], private companies, nongovernmental organizations, and researchers explored these new channels to reach people who were otherwise cut off from health services.

Chatbots have been designed to provide a range of health-related services, from information sharing [6] to offering support for mental well-being [7,8], and diagnostic tools to assist health care workers [9]. They have been designed for a range of subgroups such as specific age-groups (eg, youth who are often considered the most likely group to accept and adopt mobile technology), people with specific diseases (eg, young people with diabetes [10]), or those in health care roles (eg, frontline health workers [11]).

While this theme issue in the *Journal of Medical Internet Research* titled “Chatbots and COVID-19” [12] focuses on chatbots related to COVID-19, the span of topics covered by these chatbots is broad. They range from vaccine hesitancy and equity in access to relevant information [13-15] to pandemic-related mental health [16-19]. A number of topics focus on health promotion interventions, such as smoking cessation [20], as well as encouraging physical activity [21] and tooth-brushing [22]. Although the topics are diverse, some common themes have emerged from these explorations, namely, the importance of considering user experience in chatbot design, the need to make chatbots inclusive, and the importance of user trust [23].

The perception of chatbots in these studies was generally positive: user experiences demonstrate that the chatbots are seen as valuable tools for managing personal health [24-26] and are considered useful in health care and clinical settings [27]. However, there was a clear paucity of robust evidence on the impact these chatbots have on health outcomes. Chatbots are a medium for delivering content, but the quality, relevance, and impact of the content itself is as essential. Not all chatbots are equal, and potential efficiency gains should not be assumed to deliver automatically. Some chatbots may potentially do more harm than good. There is an urgent need for more implementation research on best-practice monitoring and evaluation of chatbots in terms of their contribution to health, as well as their strengths and weaknesses compared to other mediums when used in different contexts or with different populations.

It is also important to not lose sight of the audience. In some cases, focusing on a chatbot at the expense of alternative approaches may lead to the marginalization of certain population groups. There are inherent access barriers created by chatbots. Most notably, of course, is the access to the mobile technology itself, which is not equally distributed globally. Although mobile phones have become ubiquitous globally, mobile data can be unreliable and expensive in many contexts and the exclusion of certain groups due to age or language restrictions, for example, can be a major challenge [28]. Nonetheless, chatbots can also be a valuable tool for increasing access by overcoming literacy barriers for some populations and some globally focused chatbots have already been developed in multiple languages. Pahola (a chatbot designed to help people reduce their alcohol consumption [3]) and Florence (a chatbot designed to help people with multitude of health concerns, including smoking cessation [4]), for example, “speak” 4 and 7 different languages, respectively.

There is reason to be optimistic about chatbots and new digital tools for health. They hold the potential to remove access barriers and engage marginalized populations. Over the past 2 decades, there has been some *hype* around these innovations, however, and excessive confidence about the potential benefits of chatbots despite a lack of evidence on their quantifiable impact on health outcomes. Furthermore, as recent debates on the role of artificial intelligence (AI) showcase, technology can trigger unintended consequences to both its users and wider society. Although chatbots are not all the same as AI—indeed, many in this series are often closer to traditional SMS text messaging than to a machine learning model—a similar awareness of context and opportunity costs is essential for their healthy development. To fully understand the benefits of health chatbots, providers must work closely with a broad spectrum of perspectives, including local communities, social scientists, anthropologists, and ethicists, to ensure that their development addresses growing concerns about digital harms [2,29]. Without adequate ethical guidance, transparency, and accountability of developers and products, there is a risk of harming users through intentional or unintentional discrimination, marginalization, or lack of privacy [29].

In May 2023, WHO officially declared the end of the COVID-19 international health emergency. Nevertheless, its consequences for health systems and digital health development will continue to reverberate for some time. A wider interest in health chatbots may have started as a stopgap, but their use and value have not ended with the pandemic. However, chatbots must adhere to the same evidence-based standards as any new tool in a public health or clinical setting. Without this robust foundation, chatbots will remain tools that are at best a nice-to-have, and at worst only considered when other options are unavailable.

## Abbreviations

AI: artificial intelligence
CDC: Centers for Disease Control and Prevention
WHO: World Health Organization
